# Aggressive middle turbinate osteoblastoma with intracranial extension: a case report

**DOI:** 10.1186/1752-1947-8-161

**Published:** 2014-05-23

**Authors:** Draško Cikojević, Zaviša Čolović, Bernarda Lozić, Marisa Klančnik

**Affiliations:** 1 Department of Othorinolaryngology, University Hospital Split, Spinčićeva 1, 21000 Split, Croatia; 2 Department of Pediatrics, University Hospital Split, Spinčićeva 1, 21000 Split, Croatia

**Keywords:** Osteoblastoma, Middle turbinate, Nasal obstruction

## Abstract

**Introduction:**

Osteoblastoma is an uncommon benign bone tumor that accounts for 1 percent of all primary bone tumors. About 30 to 40 percent of all osteoblastoma cases involve the spine. Osteoblastoma involving the nasal cavity is rare, with only 11 reported cases in the English-language literature, while only four cases of turbinate osteoblastoma have been described.

**Case presentation:**

We report an unusual case of middle turbinate osteoblastoma associated with right-sided nasal obstruction and severe headache in a 14-year-old Caucasian girl. The tumor involved the right middle turbinate, complete anterior and incomplete posterior ethmoidal cells, and the frontal sinus ostium. Cribriform lamina was, in the most part, consumed by the tumor growth, while the skull base was mostly of normal bone structure.

**Conclusions:**

To the best of our knowledge, this is the first case of middle turbinate osteoblastoma with intracranial spread. Surgical treatment is the only therapeutic option for osteoblastoma.

## Introduction

Osteoblastoma is an uncommon benign bone tumor that accounts for 1 percent of all primary bone tumors [1,2]. The term 'benign osteoblastoma' was independently proposed by Jaffe and Lichtenstein to define a vascular osteoid and bone-forming tumor containing numerous osteoblasts and rich vascularized delicate fibrous stroma with a benign appearance [3,4].

About 30 to 40 percent of all osteoblastoma cases involve the spine [5,6]. The most common area of involvement is the cervical spine (20 to 40 percent), followed by the lumbar spine. Approximately 10 to 15 percent arise within the bones of the craniofacial skeleton [7,8]. The most common location is the mandible, followed by the maxilla. Osteoblastoma involving the nasal cavity is rare, with only 11 reported cases in the English-language literature [9,10], while only four cases of turbinate osteoblastoma have been described [11-13]. Most nasal cavity cases originate from the ethmoid sinus and spread to involve the nasal cavity. Osteoblastomas usually occur in patients aged <30, with a peak incidence in the second decade of life, age range 3 to 78 years. The tumor shows a predilection for male gender (M [male]:F [female], 2.5:1). Most osteoblastomas measure between 2 and 5cm, and are solid and dark red due to rich vascularity. The tumors have poorly defined margins.

**Figure 1:**
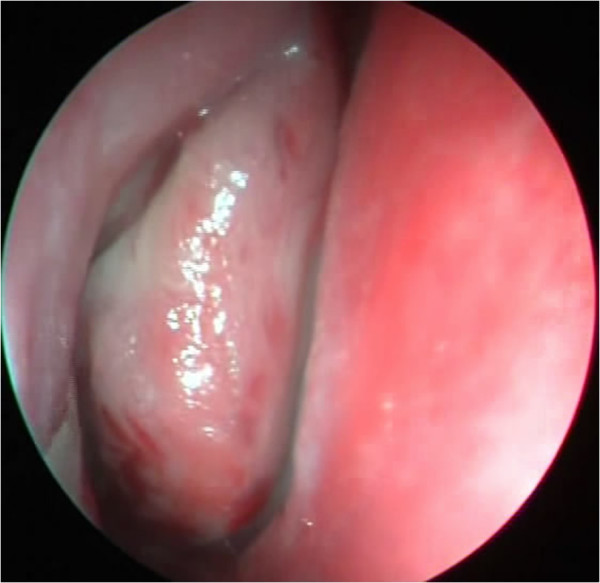
Osteoblastoma of the right middle turbinate.

Differential diagnosis of osteoblastoma includes osteoma, osteoid osteoma, chronic osteomyelitis (Brodie's abscess) and osteosarcoma. The treatment of osteoblastoma is principally surgical excision. *En bloc* resection is usually curative, however, curettage results in a local recurrence rate of approximately 20 percent. Malignant transformation of osteoblastoma to osteosarcoma is exceptionally rare (<1 percent).We report an unusual case of middle turbinate osteoblastoma associated with a right-sided nasal obstruction and severe headache in a 14-year-old Caucasian girl (Figure 1). To the best of our knowledge, this is the first case of middle turbinate osteoblastoma with intracranial spread.

## Case presentation

**Figure 2:**
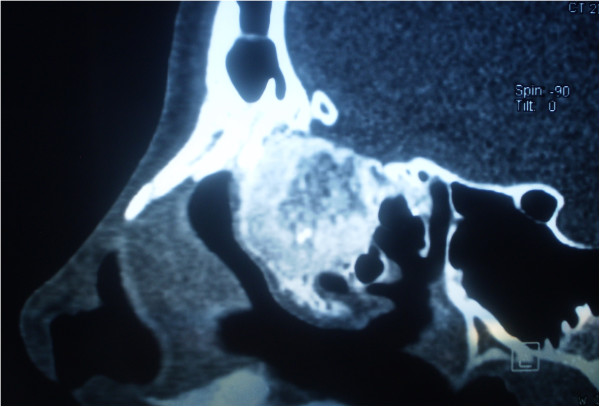
Sagittal multislice computed tomography showed a clearly delineated, heterogeneous (hyperechoic) tumor growth with inhomogeneous central portion and marginal mineralization.

**Figure 3:**
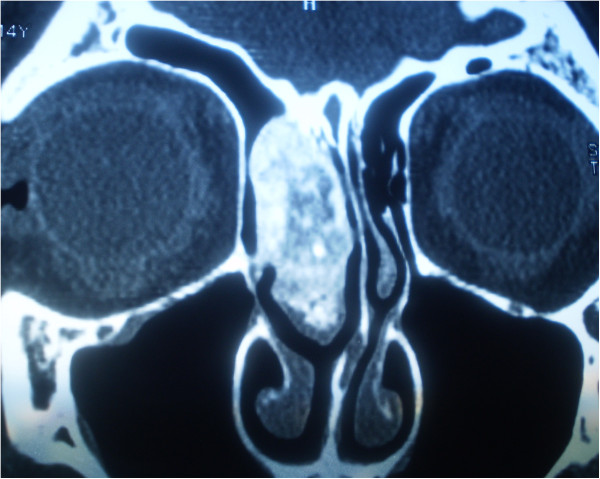
Coronal multislice computed tomography image of osteoblastoma.

**Figure 4:**
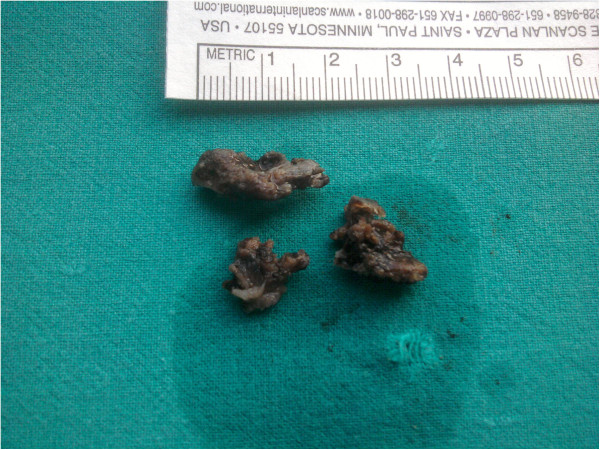
Excised specimen.

**Figure 5:**
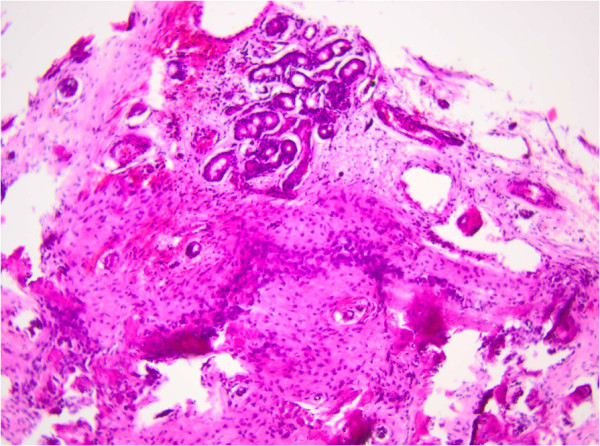
Pathohistologic finding of osteoblastoma, hemotoxylin and eosin x 60.

**Figure 6:**
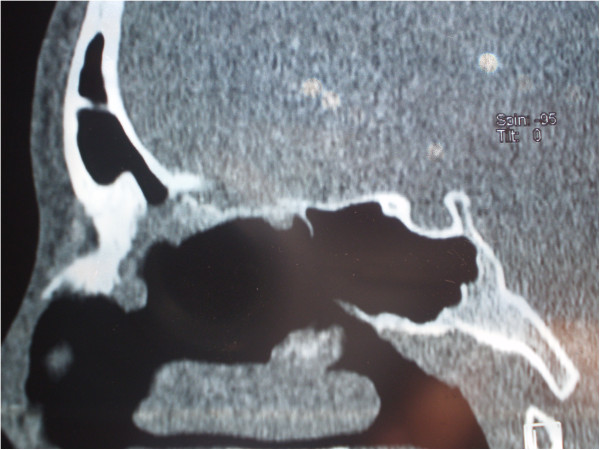
Sagittal multislice computed tomography one year postoperatively.

**Figure 7:**
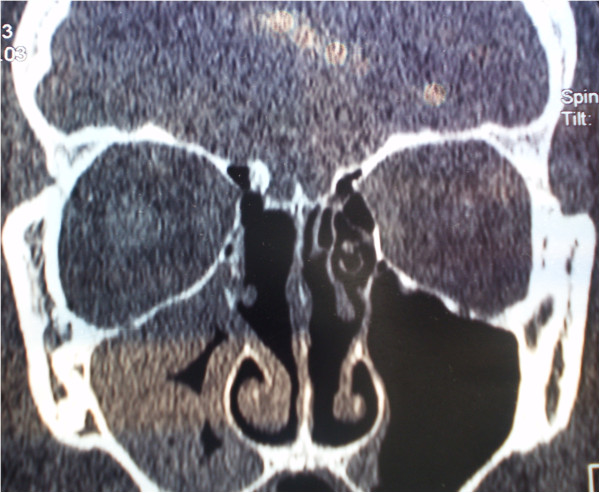
Coronal multislice computed tomography one year postoperatively.

**Figure 8:**
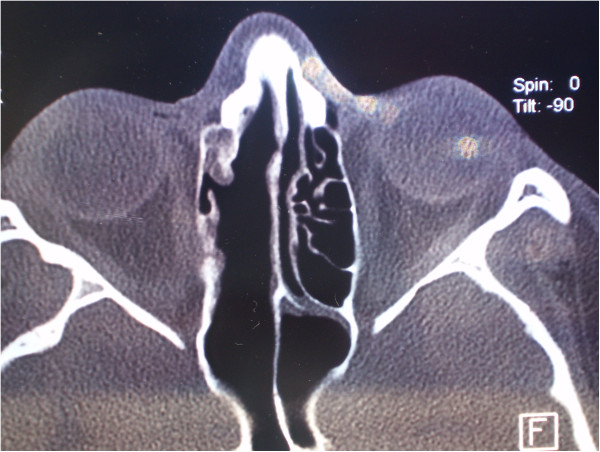
Axial multislice computed tomography one year postoperatively.

A 14-year-old Caucasian girl presented with a right-sided nasal obstruction and severe headache, which was most pronounced in the right forehead region. Our patient reported a headache persisting for three years, however, in the past three months, its intensity prevented her from attending school. In addition to difficult breathing on the right turbinate, she reported anosmia and occasional epistaxis on the same nostril. On several occasions, our patient was seen by a pediatrician, who considered her headaches to be related to puberty. When her headache turned so severe to prevent her attending school, a magnetic resonance imaging (MRI) scan of her brain was recommended to reveal a tumor of the right middle turbinate. A multislice computed tomography (MSCT) scan of her paranasal sinuses, taken for better visualization of the bone structure, showed a clearly delineated, heterogeneous (hyperechoic) tumor growth, which corresponded to the finding of a bone tumor with a inhomogeneous central portion and marginal mineralization (Figures 2 and 3). The tumor involved the right middle turbinate, complete anterior and incomplete posterior ethmoidal cells, and the frontal sinus ostium. Cribriform lamina was, in the most part, consumed by the tumor growth, while the skull base was mostly of normal bone structure. Lamina papyracea was free and clearly demarcated from the tumor. There was septum shift to the left, without septum perforation. The tumor was removed *in toto* by endoscopic technique (Figure 4) and pathohistological analysis showed the osteoblastoma (Figure 5). As it was a bone tumor, it could not be excised *en bloc*, instead, the tumor was first cut by a bur in the region of the middle turbinate base, then the rest of the tumor was removed by diamond bur abrasion of the bone with removal of the most part of the cribriform lamina. On endoscopy, there was a clear margin between the healthy bone, which was white and compact, and osteoblastoma, which was of a honeycomb structure, reddish and more vascularized. Postoperatively, our patient denied headache, breathed normally through her nose and attended school regularly. But the six-month follow-up computed tomography (CT) scan showed a recurrence and we had to do an *en bloc* resection of the tumor. The CT scan one year later showed normal findings without recurrence and our patient did not have her headache any more (Figures 6, 7 and 8).

## Discussion

Osteoblastoma is a rare bone tumor and accounts for approximately 1 percent of all primary bone tumors. On differential diagnosis, it is important to differentiate osteoblastoma from osteoma, osteoid osteoma and osteosarcoma.

Osteomas are benign, slow-growing tumors that rarely occur in children. They usually arise on the surface of the cranial vault, jaw, paranasal sinuses and orbit [14]. Osteomas are often asymptomatic and are frequently an incidental finding. They are round, oval, white, well circumscribed and attached to the underlying bone by a broad base, or occasionally by a small stalk. On bisection, they are dense or sclerotic, with narrow (compact type) or prominent (spongiotic) intertrabecular spaces. Recurrences are very rare, even in incompletely excised lesions. Malignant transformation has not been reported.

Osteoid osteoma is 1 to 2cm in diameter, and morphologically similar lesions larger than 2cm are classified as osteoblastomas [1,5]. It accounts for approximately 12 percent of benign bone tumors and, similar to osteoblastoma, it predominantly affects children and young adults, particularly female. Clinically, osteoid osteoma most commonly occurs in the long bones (for example, femur, tibia). The lesions cause night pain that is relieved with nonsteroidal anti-inflammatory drugs (NSAIDs). On radiography, osteoid osteomas exhibit a central lucency (the nidus) with patchy mineralization located centrally. Osteoid osteoma may be managed nonsurgically with NSAIDs.

Osteosarcoma is the most common primary malignant tumor of bone. In the craniofacial region, osteosarcoma mostly affects jaw bones, with approximately 6 percent of all [15]. The majority of patients are older than 30 years of age. On radiography, osteosarcoma shows a poorly defined destructive lesion that can be sclerotic, lytic or mixed.

Osteoblastoma occurs predominantly in young adults. It develops most frequently in the posterior element of the vertebrae and in long bone metaphyses. Patients present with a painful mass and craniofacial tumors may produce headache, tooth impaction and epistaxis. The pain does not get worse at night and is less likely to be relieved with NSAIDs. Most osteoblastomas measure between 2 and 5cm and are solid, dark red, due to the rich vascularity, tan-white and gritty. Some tumors have more poorly defined margins. On radiography, osteoblastomas arise in the medullary cavity or on the surface of the bone. The intralesional mineralization is very variable and may be minimal or extensive, but is greatest centrally. The intertrabecular space is filled with richly vascular loose connective tissue. The treatment of osteoblastoma is principally surgical excision. *En bloc* resection is usually curative, however, curettage results in a local recurrence rate of approximately 20 percent. Malignant transformation of osteoblastoma to osteosarcoma is exceptionally rare (<1 percent).

Reliable preoperative diagnosis is of utmost importance, which is rather difficult in bone tumors because cytologic biopsy is impossible due to the hard tumor structure. Biopsy can be done in osteoblastoma, however, hemorrhage may occur because of rich tumor vascularization. It is crucial to differentiate osteoid osteoma from osteoblastoma because the former can be managed by medicamentous therapy (NSAIDs), whereas surgical treatment is the only therapeutic option for osteoblastoma.

## Conclusions

To the best of our knowledge, this is the first case of middle turbinate osteoblastoma with intracranial spread. Surgical treatment is the only therapeutic option for osteoblastoma.

## Consent

Written informed consent was obtained from the patient’s parents for publication of this case report and any accompanying images. A copy of the written consent is available for review by the Editor-in-Chief of this journal.

## Competing interests

The authors declare that they have no competing interests.

## Authors’ contributions

DC was the surgeon, participated in the sequence alignment and drafted the manuscript. ZC participated in the sequence alignment. BL participated in the design of the study and performed the statistical analysis. MK conceived of the study, and participated in its design and coordination and helped to draft the manuscript. All authors read and approved the final manuscript.
